# Mitotically active cellular fibroma of the ovary: A case report

**DOI:** 10.4274/tjod.11298

**Published:** 2015-03-15

**Authors:** Nuri Yıldırım, Bahadır Saatli, Fatma Akalın, Çağnur Ulukuş, Funda Obuz, Uğur Saygılı

**Affiliations:** 1 Dokuz Eylül University Faculty of Medicine, Department of Obstetrics and Gynecology, İzmir, Turkey; 2 Dokuz Eylül University Faculty of Medicine, Department of Pathology, İzmir, Turkey; 3 Dokuz Eylül University Faculty of Medicine, Department of Radiology, İzmir, Turkey

**Keywords:** Mitosis, pelvic mass, cellular fibroma

## Abstract

Fibromas are classified in a spectrum from fibromas to fibrosarcomas according to the number of mitosis they include. Malignant fibrosarcomas which have aggressive pattern show higher mitotic activity and nuclear atypia. Cellular fibromas with less than 4 mitotic figures under 10 high power fields (HPF) are benign. “Mitotically active cellular fibromas” that are classified between the cellular fibromas and fibrosarcomas, have ≥4 mitotic figures in 10 HPF but do not have nuclear atypia. A very few cases of mitotically active cellular fibromas have been reported in the literature. In this report, we present the case of mitotically active cellular fibroma in a patient who applied to our clinic with the complaint of pelvic mass.

## INTRODUCTION

Cellular fibroblastic tumors are classified as fibroma or fibrosarcoma. Cellular fibromas include less than 4 mitoses (<4MF/10HPF), have normal nucleus and benign feature. Fibrosarcomas have more than 4 mitoses, display nuclear atypia and they are malignant^(1)^. There are few cases with benign features but high mitosis count, named as “mitotically active cellular fibroma”^(2,3,4,5,6)^. These cases have more than four mitotic figures but behave as benign. Clinical outcome is different from fibrosarcoma which is an aggressive disorder. In this report, a case of mitotically active cellular fibroma (MACF) will be discussed.

## CASE

**Figure 1:**
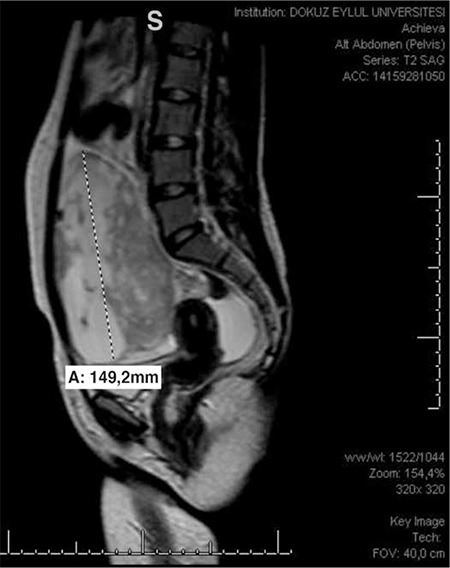
Magnetic resonance imaging of the mass

**Figure 2:**
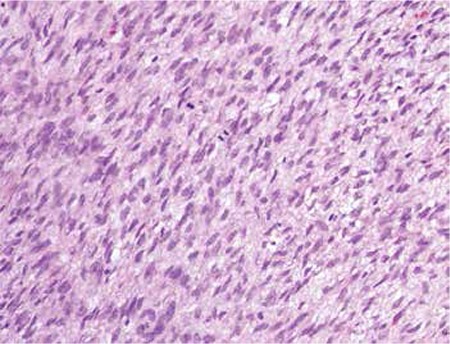
Microscopic appearance of mitotically active cellular fibroma (MACF) (40x)

A twenty-four-year old single woman presented with abdominal distention and a palpable abdominal mass. On physical examination, a pelvic mass measuring 15 cm in diameter was palpated up to the level of the umbilicus. In abdominal ultrasonography, a 14 cm left adnexal mass which had solid-cystic pattern was observed. Magnetic resonance imaging (MRI) revealed increased signal intensity in the fat-suppressed T1A sequences of the solid components of the lesion and probable hemorrhagic lesions were considered. In the diffusion-weighted series, low signal intensity was observed in the solid components (Figure 1). Serum CA125 level was high, which was 297 U/ml, and other markers, such as LDH, hCG and AFP were normal. The patient was operated with the suspicion of malignancy. In the abdominal evaluation during laparotomy, a left adnexal 15 cm mass that had cystic-solid parts with smooth surface was detected. There were no ascites. There were dense adhesions between the mass and the sigmoid colon. During adhesiolysis, the cystic part was ruptured. Cystectomy was performed due to the age and fertility desire of the patient. Frozen section was reported as benign. The adhesive tissue over the sigmoid colon and the bladder peritoneum was also excised. Contralateral ovary, uterus and other visceral surfaces seemed normal. Final pathology result was reported as fibromatous tumor of the ovary. It was supported with the immunohistochemical positivity of ER, PR and WT1. In addition, there were ischemic necrosis, cellularity and 4 mitoses under 10 HPF, but there were no nuclear atypia. Therefore, the final diagnosis was confirmed as mitotically active cellular fibroma. The adhesive tissue over the sigmoid colon and the bladder peritoneum was also diagnosed as the same. ER, PR, calretinin, inhibin-alfa, WT-1, h-caldesmon and CD10 were positive; pancytokeratin, EMA, CD34, desmin, and S-100 were negative; CD56 and actin were focal weakly positive. Ki-67 index was 8% (Figure 2).

There were no intra- or post-operative complications and she was discharged on the second day following the surgery. She is still followed up by our clinic and there is no recurrence.

## DISCUSSION

Malignant sex cord stromal tumors are the second most common ovarian tumors after epithelial ovarian tumors. Categorized within this group, fibrosarcomas which have excess in number of cellular mitosis and with nuclear atypia are distinguished from fibroma. Prat and Scully made the first analysis on this issue and reported that tumors with four or more mitoses are named as fibrosarcoma^(7)^. However, some case reports in the literature and the widest study on this subject which was reported by Irving et al. claimed that tumors with four and more mitoses, but without nuclear atypia should not be regarded as fibrosarcoma^(4)^. These cases should be named as “mitotically active cellular fibroma” (MACF).

Irving et al. reviewed 40 MACF and 35 cellular fibroma cases^(4)^. The median age of the patients in MACF group was 41 years. Our patient, who was 24 years old, is one of the youngest patients in the literature. The most important symptom is pelvic mass, as in our case. Abdominal pain, acute abdomen due to torsion, dysfunctional uterine bleeding, and ascites can be other symptoms in a decreasing frequency. Incidentally detected cases on physical examination, ultrasound, or intraoperatively (caesarean section, etc.) have been reported. Meigs syndrome with pleural effusion was found in a patient^(4)^. In the literature, 2 cases mimicking epithelial ovarian cancer with CA125 elevation have been reported^(8)^. In our case, CA125 level was above the normal limits; 297 U/mL.

The average size of MACF has been reported to be 9.4 cm^(4)^. In our case, the tumor size (15 cm) is larger than the tumors reported in the literature. Extraovarian disease has also been reported in some patients. This involvement is usually in the form of adhesions to other genital organs (fallopian tube, uterus) or on the peritoneal surface of pelvic sidewall, it can also be on the omentum or bowel mesentery. In our case, the tumor was found in the mesentery of the sigmoid colon and in adhesions to the bladder peritoneum.

In the microscopic evaluation, MACF includes more cellularity compared to a cellular fibroma but does not contain nuclear atypia (bland nuclei). The median mitosis count was found to be 6.7 although 19 mitoses were reported under 10 HPF^(4,7)^. Prat and Scully reported no recurrences in mild nuclear atypia group and in 66% (4/6) of moderate nuclear atypia patients^(7^). One of the remaining 2 patients died due to recurrence, 2.75 years after the diagnosis. Other patient with recurrence died from non-tumoral causes. Once more, it is necessary to re-emphasize that patients with recurrence have moderate nuclear atypia. Irving et al. reported 4.75-year follow-up of 18 MACF cases with 3 extraovarian involvement and evidence of recurrence was observed in none of the patients^(4)^. Kaku et al. reported a case of MACF with 10 mitoses under 10 HPF without any recurrence in one-year follow-up^(9)^.

The only study that reported recurrence in patients with MACF in the literature was published by Bucella et al.^(10)^. This case was a 65-year-old woman and recurrence was seen in the Douglas pouch 5 years after the operation. Adjuvant tamoxifen (20 mg/day) was started after surgical resection and no recurrence was reported at six months follow-up.

In these cases, fertility-sparing surgery is of great importance due to average age of occurrence of the disease. Therefore, the correct diagnosis of the disease has the most important role in protecting the patient’s fertility. Due to subjective evaluation of the cellular atypia, diagnosis should be correlated with clinicopathologic and immunohistochemical data in order to perform fertility-sparing surgery and to prevent initiation of adjuvant treatment.

As a result, the clinical behavior cannot be predicted accurately and local recurrence can be seen in these tumors. Thus, it is indicated that they should be regarded as a low malignant potential tumor. Therefore, morphological findings should be correlated with intraoperative and other clinical/imaging findings and close monitoring of patients is recommended.
